# Ag-Modified In_2_O_3_ Nanoparticles for Highly Sensitive and Selective Ethanol Alarming

**DOI:** 10.3390/s17102220

**Published:** 2017-09-27

**Authors:** Jinxiao Wang, Zheng Xie, Yuan Si, Xinyi Liu, Xinyuan Zhou, Jianfeng Yang, Peng Hu, Ning Han, Jun Yang, Yunfa Chen

**Affiliations:** 1 State Key Laboratory of Multiphase Complex Systems, Institute of Process Engineering, Chinese Academy of Sciences, Beijing 100190, China; wangjinxiao@stu.xjtu.edu.cn (J.W.); zheng163xie@163.com (Z.X.); zhouxinyuan14@mails.ucas.ac.cn (X.Z.); pengh@bjut.edu.cn (P.H.); chenyf@ipe.ac.cn (Y.C.); 2 School of Metallurgical Engineering, Xi’an University of Architecture and Technology, Xi’an 710055, China; 3 Beijing ChenJingLun High School, Beijing 100101, China; siyuan0517@126.com (Y.S.); siww@x263.net (X.L.); 4 State Key Laboratory for Mechanical Behavior of Materials, Xi’an Jiaotong University, Xi’an 710049, China; yang155@mail.xjtu.edu.cn

**Keywords:** In_2_O_3_ nanoparticles, Ag modification, heterojunction, high response, ethanol sensing

## Abstract

Pure In_2_O_3_ nanoparticles are prepared by a facile precipitation method and are further modified by Ag. The synthesized samples are characterized by scanning electron microscopy, transmission electron microscopy, energy dispersive X-ray spectroscopy, X-ray diffraction, Raman and UV-Vis spectra. The results show the successful heterojunction formation between Ag and In_2_O_3_. Gas sensing property measurements show that the 5 mol % Ag-modified In_2_O_3_ sensor has the response of 67 to 50 ppm ethanol, and fast response and recovery time of 22.3 and 11.7 s. The response is over one magnitude higher than that of pure In_2_O_3_, which can be attributed to the enhanced catalytic activity of Ag-modified In_2_O_3_ as compared with the pure one. The mechanism of the gas sensor can be explained by the spillover effect of Ag, which enhances the oxygen adsorption onto the surface of In_2_O_3_ and thus give rise to the higher activity and larger surface barrier height.

## 1. Introduction

Each year, a large number of traffic accidents are triggered by drunk driving, causing serious casualties and property losses. Therefore, strict regulations have been made by traffic management organizations to limit drunk driving, and many kinds of methods such as electrochemical sensors have been used for the rapid detection of alcohol concentration in exhaled breath. Due to their low-cost and simple operation, semiconductor metal oxide (SMOX) gas sensors have gained wide attention [1,2,3,4,5,6,7]. In_2_O_3_ is a typical n-type semiconductor with a wide band gap of about 2.8 eV, and has been used in gas sensors due to its high specific surface areas [8,9,10,11]. However, pure In_2_O_3_ gas sensors have inadequate sensitivity to low concentrations of ethanol for making better judgments about whether the subject is inebriated. For example, according to the Chinese standard (GB 19522-2010), there are two types of penalties for drunk driving: drunk driving (breath alcohol concentration exceeds 177 ppm) and driving after drinking (breath alcohol concentration is 44~177 ppm). Similar constraints have been recommended by World Health Organization and many other countries in the world. Consequently, the sensitivity of In_2_O_3_ should be improved to a detection limit of <44 ppm ethanol in order to screen drinkers quickly.

Commonly, the sensing properties of In_2_O_3_ are tailored by morphology control, structure modification, and decoration with metal oxides to for heterojunction [12,13,14,15,16], as well as modified with noble metals [17,18] in the matrix surface area. For example, hierarchical In_2_O_3_ microbundles and flowers were prepared, showing a response of 11–14 to 50 ppm ethanol [12,15]. Anand [18] utilized Tb to dope In_2_O_3_ nanorods, which showed a high response of 40 to 50 ppm ethanol. Meanwhile, noble metals are commonly used as surface modifiers to enhance the response of MOX sensors. For instance, Pd can enhance the sensitivity of In_2_O_3_ to ethanol with a response of 50 to 100 ppm [19]. However, Au nanoparticle decoration on In_2_O_3_ can only obtain the response of 6.5 to 100 ppm ethanol [17]. Therefore, the performance enhancement of In_2_O_3_ gas sensors should be further improved to obtain a high response to ethanol. In the meantime, the resistance of the gas sensors should be in the kΩ level in order to be easily integrated with electronic circuits. In contrast, complicated circuit design should be adopted and a higher cost is thus unavoidable. Therefore, there exist trade-offs between the parameters of a gas sensor, including sensitivity, response/recovery time, resistance level, etc.

In this study, pure In_2_O_3_ were prepared by a simple precipitation method, and were then modified by Ag to form a surface heterojunction. The gas sensing properties of pure In_2_O_3_ and Ag-modified In_2_O_3_ were compared and the catalytic performances were evaluated. The results show that Ag can greatly improve the ethanol sensing property of In_2_O_3_, demonstrating a response of 67 to 50 ppm at a sensor resistance of about 10 kOhm. The mechanism is attributable to the enhanced ethanol catalytic property of In_2_O_3_ by Ag with a significant spillover effect.

## 2. Materials and Methods

### 2.1. Synthesis of Ag-Modified In_2_O_3_ Nanoparticles

The 0.5 mol·L^−1^ NH_3_·H_2_O solution was added dropwise into 10 mL 1 mol·L^−1^ InCl_3_ aqueous solution at 60 °C. After the pH was adjusted to 8.5, the solution was then heated for about 30 min and then cooled down to room temperature. Subsequently, the precipitate was rinsed, dried, and calcined at 600 °C for 2 h to obtain the pure In_2_O_3_ product.

In Ag modification, the In_2_O_3_ was mixed uniformly with 0.03 mol·L^−1^ AgNO_3_ solution. The molar ratio (Ag/In) was 1, 5, 10, and 20 mol %, respectively. After water was completely evaporated at 100 °C, the mixture was calcined at 500 °C for 2 h in a tube furnace and then cooled down to room temperature. Eventually, the product was obtained after it was ground with an agate mortar.

### 2.2. Characterization

The crystalline phase determination was carried out by X-ray powder diffraction (XRD, Panalytical X’pert Pro, Alemlo, The Netherlands, Cu-Kα radiation of λ = 0.15406 nm, 40 kV, 40 mA). The morphologies and energy dispersive X-ray spectra (EDS) were characterized by a field-emission scanning electron microscope (FE-SEM, JEOL JSM-6700F, Tokyo, Japan 5 kV, 10 μA). The microstructures were investigated by high-resolution transmission electron microscopy (HRTEM, JEOL JEM-2010F, Tokyo, Japan, 200 kV, 100 μA). The optical properties were test by Raman (532 nm argon laser, Invia-Reflex 20 mW, Renishaw, London, Britain) and UV-Vis spectra (300UV-vis spectrophotometer, Thermo Scientific, Waltham, MA, USA). The catalytic evaluation of ethanol was analyzed by using a gas chromatograph (Shimadzu GC-2014, Tokyo, Japan).

The gas sensing performance of the sensors was measured by using a WS-30A Gas sensor test system (Zhengzhou Winsen Electronics Technology Co. Ltd., Zhengzhou, China) according to previous studies [20,21,22]. The samples were ultrasonically dispersed with a suitable amount of ethanol. Then, the mixture was extracted with a micro-syringe and dribbled onto the surface of a substrate with a pair of Au electrodes on both ends. A Ni-Cr heating wire went through the substrate to serve as a heating filament, and the operating temperature was controlled by tuning the heating voltage. The gas response is defined as the ratio of R_a_/R_g_, where R_a_ and R_g_ are the resistances of the sensor in the air and tested gas, respectively.

## 3. Results

### 3.1. Synthesis and Characterization

**Figure 1:**
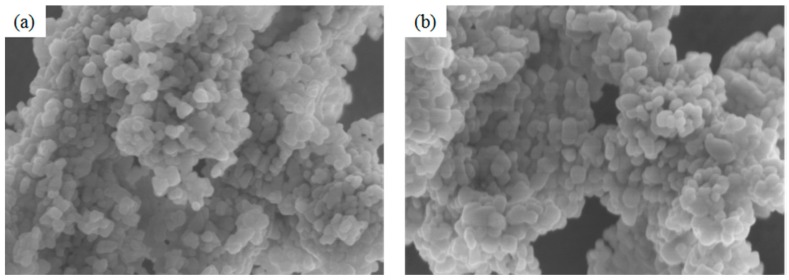
SEM images of (**a**) pure and (**b**) 5 mol % Ag-modified In_2_O_3_ nanomaterials.

**Figure 2:**
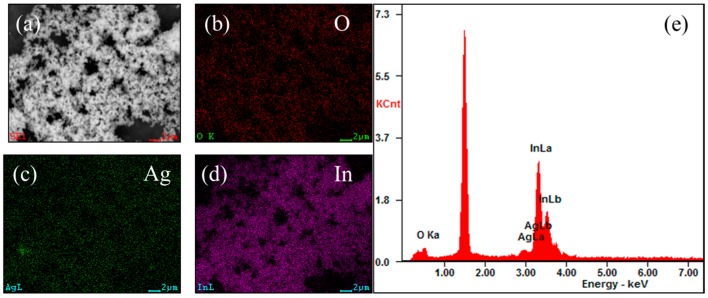
SEM images, element mapping and EDS spectrum of 5 mol % Ag-modified In_2_O_3_ nanomaterials. (**a**) SEM image; (**b**) O; (**c**) Ag; (**d**) In mapping; and (**e**) EDS spectrum.

The SEM images of In_2_O_3_-based nanomaterials (Figure 1) demonstrate that the pure In_2_O_3_ and 5 mol % Ag-modified In_2_O_3_ have similar morphologies of nanoparticles with diameters of about 50 nm. It is difficult to distinguish Ag nanoparticles from the image due to the inadequate magnification. Then EDS elemental analysis was carried out with the mapping and spectra of 5 mol % Ag-modified In_2_O_3_ nanomaterials, as shown in Figure 2. It can be seen clearly that the Ag element is identified in the EDS mapping and the spectrum. We can calculate the Ag concentration to be 4.4 mol %, which is similar to the Ag concentration (5 mol %) in the experimental design.

**Figure 3:**
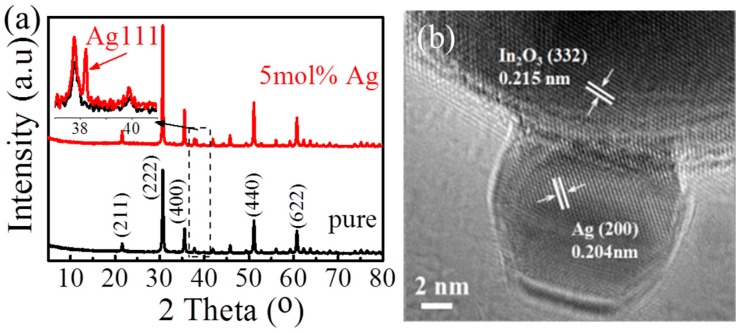
(**a**) XRD patterns of pure and 5 mol % Ag-modified In_2_O_3_ nanomaterials, and (**b**) HRTEM images of 5 mol % Ag-modified In_2_O_3_ nanomaterials.

X-ray diffraction (XRD) is then used to determine the crystal structure of the products as shown in Figure 3a. It is clear that the peaks of pure In_2_O_3_ are (211), (222), (400), (440), (620), which can be indexed to the cubic structure of In_2_O_3_ (PDF No. 01-071-2194). This shows that the pure In_2_O_3_ has good crystallinity. The diffraction peak of Ag (111) can be found clearly at 5 mol % Ag-modified In_2_O_3_ [10,23,24]. To further identify the Ag nanoparticles, HRTEM images were obtained, as shown in Figure 3b. It is clearly seen that the In_2_O_3_ particles are approximately 50 nm and the smaller Ag nanoparticles (about 10 nm) are pinned on the In_2_O_3_ surface (Figure 3b). Furthermore, the lattice spacing of 0.215 nm corresponds to the In_2_O_3_ (332) plane, and the lattice spacing of 0.204 nm corresponds to the Ag (200) plane. These results indicate that the In_2_O_3_ has been successfully modified by Ag, forming a heterostructure.

**Figure 4:**
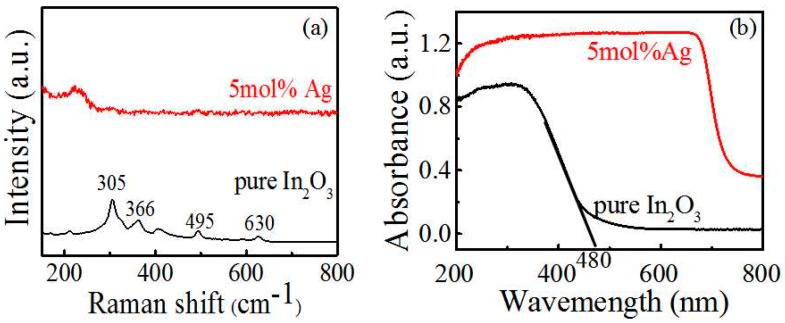
(**a**) Raman spectra and (**b**) UV-Vis spectra of pure and 5 mol % Ag-modified In_2_O_3_ nanomaterials.

The structural and optical properties are then characterized by Raman and UV-Vis spectra as shown in Figure 4. The peaks at around 305, 366, 495, and 630 cm^−1^ in Figure 4a fit well with the bcc-structured indium oxide [25]. Also, the peak around 305 cm^−1^ belongs to the bending vibration of InO_6_, the peak around 366 cm^−1^ belongs to the stretching vibration of In-O-In, and those around 495 and 630 cm^−1^ are attributed to the stretching vibration of lnO_6_. We found that the Raman peaks nearly disappeared with Ag addition. The Ag may lead to a strong fluorescence and thus the stretching vibration and bending vibration are greatly reduced [26]. The UV-Vis absorption spectra of the pure and Ag-modified In_2_O_3_ are clarified in Figure 4b. The pure In_2_O_3_ exhibits light absorption at about <480 nm, from which we can calculate the band gap of pure In_2_O_3_ to be about 2.8 eV according to the Kubelka-Munk equation [24]. This result is basically the same as that in previous studies [8,11]. However, the absorption edge is greatly extended to the visible light region of about 700 nm, which is probably due to the surface plasmon response absorption of Ag nanoparticles as reported in the literature [24]. The Ag plasmonic absorption is dependent on the diameter of the nanoparticles (red shift with increased diameter) [27]; therefore, the absorption in the 400–700 nm range in this study showed the large Ag size distribution [25].

### 3.2. The Gas Sensing Properties

**Figure 5:**
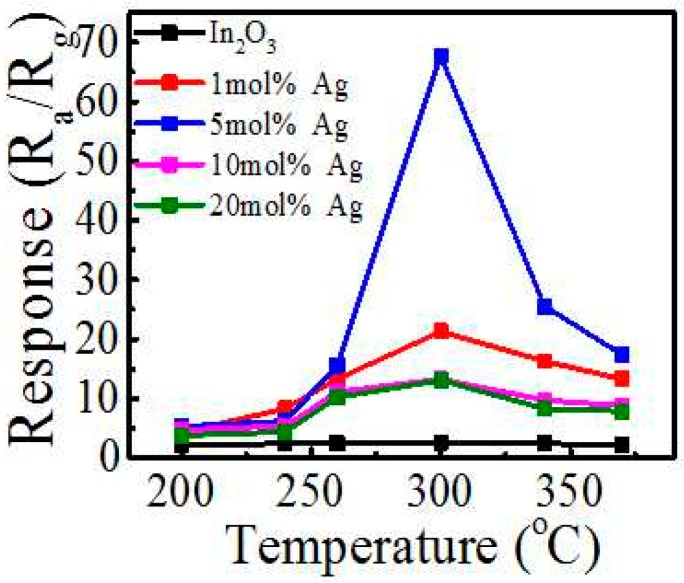
Responses vs operating temperatures of Ag-modified In_2_O_3_ nanomaterials.

The gas sensing properties of pure and Ag-modified In_2_O_3_ are measured by static method. The optimum operating condition is firstly investigated for ethanol detection, as shown in Figure 5. Commonly, at low temperatures, less active sites at the semiconductor surface lead to the insufficient reaction of the target gas. The target gas interacts more easily with the sensors at increased work temperatures, resulting in the response improvement. Nonetheless, upon further increasing the working temperature, the charge-carrier concentration and the conductivity increase and the Debye length decreases [14], which leads to the decrease of the response. Figure 5 demonstrates the response of pure and Ag-modified In_2_O_3_ under the different operating temperatures for 50 ppm ethanol. The response values increase first and then decrease with the rise of the operating temperature, and reach the maximum at 300 °C for all samples. We also tried to tailor the In_2_O_3_ nanoparticle size by precipitating In(OH)_3_ at varied temperatures of 40 and 80 °C in order to tune the gas sensing property. The 40 °C precipitated sample had similar diameter as those calcined at 60 °C, as well as a similar gas sensing performance. However, if the temperature increased to 80 °C, limited precipitate and product were obtained due to the relatively higher solubility of the In(OH)_3_ at higher temperatures. Though the particle size is a bit smaller (~30 nm), the gas response only increased from 2.6 to 3.5 to 50 ppm ethanol, showing the limited tailoring effect of the gas performance by particle size. On the other side, the response of 5 mol % Ag-modified In_2_O_3_ exhibited a maximum significantly higher than any other samples at 300 °C. The response is 67 to 50 ppm ethanol, which is one of the highest in the literature, as shown in the comparison in Table 1, demonstrating the significant response improvement of In_2_O_3_ by Ag surface modification. In the meantime, the resistance of the gas sensor is in the level of 10 kΩ, which would be favorable for easily integration with interfacing electronic circuits.

**Figure 6:**
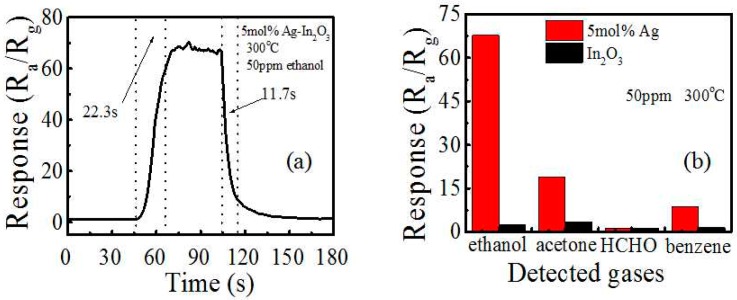
(**a**) The response-recovery characteristics of the 5 mol % Ag-modified In_2_O_3_ sensor to 50 ppm ethanol at 300 °C; (**b**) the response of the 5 mol % Ag-modified In_2_O_3_ sensor to 50 ppm of different gas concentrations at 300 °C.

To further explore the sensing property, response curve, and selectivity, responses to other interfering gases are measured in Figure 6. Figure 6a presents the response and recovery behaviors of the 5 mol % Ag-modified In_2_O_3_ sensor. The response time can be defined as the time needed to reach 90% of its saturated pulse height, while the recovery time is the time needed for the pulse to reach 10% from its base line. We found that the response-recovery times are 22.3 s and 11.7 s, respectively. In the meanwhile, the selectivity of the 5 mol % Ag-modified In_2_O_3_ sensor is evaluated by exploring the response of 50 ppm interfering gases included acetone, benzene, and HCHO at 300 °C (Figure 6b). It is observed that the Ag-modified In_2_O_3_ has a far higher response to ethanol than those to HCHO and benzene at the same concentrations, and even has a more than three times higher response to acetone. Those gases are typical pollutants detected in a car at the ppb to sub-ppm levels, which are released by the decorations and thus are the major interfering gases for ethanol detection. As ethanol from drinkers are tens of ppm in concentration, which is larger than those interfering gases, and the response to ethanol is also larger, the 5 mol % Ag-modified In_2_O_3_ sensor thus has high selectivity for ethanol detection.

**Figure 7:**
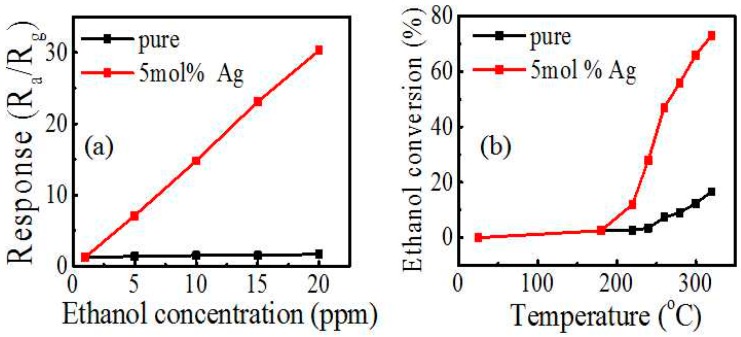
(**a**) Response change of the pure and 5 mol % Ag-modified In_2_O_3_ sensors to different ethanol concentrations at 300 °C; (**b**) ethanol conversion of the pure and 5 mol % Ag-modified In_2_O_3_ sensors as a function of reaction temperature over catalysts.

Figure 7a compares the response of pure and 5 mol % Ag-modified In_2_O_3_ under lower ethanol concentrations (1–20 ppm). It is clear that the response values tend to have an approximately linear relationship with the concentration. More importantly, the response of Ag-modified In_2_O_3_ is far higher at all concentrations. Generally, the detection limit is set at the concentration with the response of 2. It can be derived from Figure 7a that the detection of pure In_2_O_3_ is >20 ppm (response 1.7), while that of 5 mol % Ag-modified In_2_O_3_ is about 1.5–2 ppm. To shed light on the origin of the enhanced response, the catalytic properties of pure and 5 mol % Ag-modified In_2_O_3_ are compared, as shown in Figure 7b. It is noticed that the conversion increases with the rising reaction temperature, and has a striking improvement with Ag modification. The enhanced catalytic activity would be the main reason why Ag modification improved the gas sensing property.

## 4. Discussion

**Figure 8:**
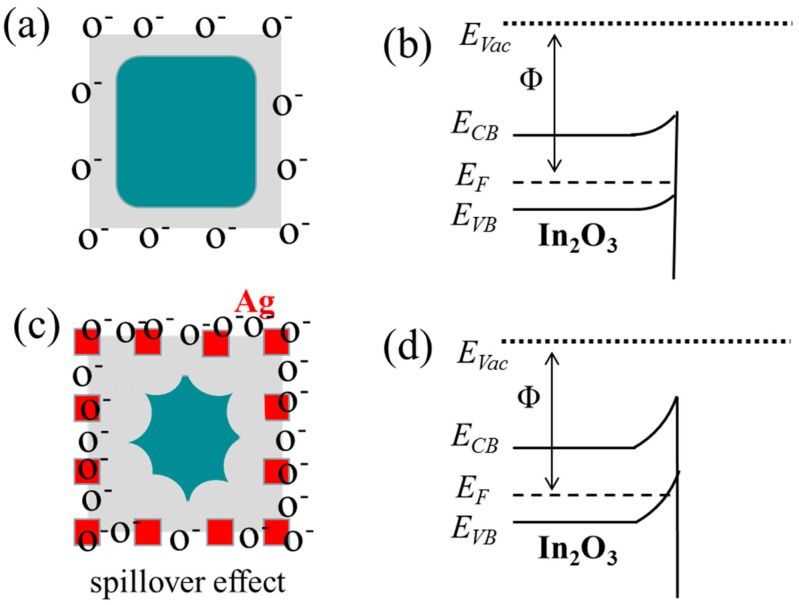
The schematic diagram (**a**) and energy band diagram (**b**) of pure In_2_O_3_; the schematic diagram (**c**) and energy band diagram (**d**) of 5 mol % Ag-modified In_2_O_3_. E_CB_ and E_VB_ are the conduction band and valence band level, E_vac_ and E_F_ represent the vacuum level and Fermi level.

On the basis of the above experimental results, the gas sensing mechanisms of pure and Ag-In_2_O_3_ sensors are schematically shown in Figure 8. The dramatic enhancement in the ethanol gas sensing performance of In_2_O_3_-based nanomaterials via modification with Ag nanoparticles can be interpreted using the electron depletion layer model [32,33]. Currently, the adsorbed oxygen ions occupy the surface area of In_2_O_3_-based nanomaterials, forming the electron depletion layer (Figure 8a). The depleted electrons and the raised electron potential (Figure 8b) by the depletion layer contribute to the high resistance of the In_2_O_3_ sensor in air. When the sensor is exposed to ethanol, the reaction between oxygen and ethanol occurs, as shown in Equation (1) [34]. Then, the trapped electrons are released to the conduction band, contributing to the thinner depletion layer and the lower resistance of In_2_O_3_ sensors.

C_2_H_5_OH _(ads)_ + 6 O^−^_(ads)_ → 2 CO_2(g)_ + 3 H_2_O + 6 e^−^ (1)

On the other side, when Ag is deposited on the surface of In_2_O_3_, there would be more oxygen adsorbed on the surface due to the well-known spillover effect [18,23,25], as shown in Figure 8c. This way, Ag modification enhances the reaction between ethanol molecules and adsorbed oxygen ions on the surface of In_2_O_3_, leading to a higher response to ethanol. Meanwhile, the more ion-adsorbed oxygen will change the energy-band structure and give rise to the potential barrier as demonstrated in Figure 8d. Therefore, the enhanced reaction with ethanol and the higher electron potential barrier will contributed to the enhance ethanol response of the Ag-modified In_2_O_3_ materials. This agrees well with the literature, confirming that Ag is a good catalyst for ethanol because Ag can effectively enhance the ethanol conversion, and suppress ethanol dehydration as well [35,36]. All these results show the potential of the Ag-modified In_2_O_3_ materials for applications in ethanol sensing.

## 5. Conclusions

In summary, In_2_O_3_ nanomaterial is successfully prepared by a facile precipitation method and is further modified by Ag as verified by EDS, HRTEM, and UV-Vis absorption characterizations. A gas sensing property test showed that the 5 mol % Ag-modified In_2_O_3_ nanomaterial has a high response of 67 to 50 ppm ethanol, a fast response and recovery time of 10–20 s, and a high selectivity over other interfering gases such as acetone, benzene, and formaldehyde. The enhanced gas sensing property is probably attributed to the higher catalytic activity to ethanol by the Ag modification as verified by the ethanol catalytic measurement. Lastly, the related mechanism is discussed in light of the spillover effect of Ag on the In_2_O_3_ surface.

## Figures and Tables

**Table 1:** Sensing properties to ethanol and acetone of the different materials in our present study and in literature studies.

Material	Ethanol	Response	T (°C)	Resistance	Reference
Ag doped In_2_O_3_	50 ppm	67	300	10 kΩ	This work
In_2_O_3_ microbundles	50 ppm	11.6	300	10 kΩ	[15]
Flower-like In_2_O_3_	100 ppm	27.6	320	180 kΩ	[12]
Au-loaded In_2_O_3_	100 ppm	6.5	140	100 kΩ	[28]
Tb doped In_2_O_3_	50 ppm	40	300	10 MΩ	[18]
Rh-loaded In_2_O_3_	100 ppm	4748	371	50 MΩ	[29]
TiO_2_ nanoparticle	20 ppm	6	400	1 GΩ	[30]
SnO_2_ nanoparticle	1000 ppm	760	350	10 MΩ	[31]
